# Effects of Paternal Predation Risk and Rearing Environment on Maternal Investment and Development of Defensive Responses in the Offspring

**DOI:** 10.1523/ENEURO.0231-16.2016

**Published:** 2016-11-17

**Authors:** Austin C. Korgan, Elizabeth O’Leary, Jessica Bauer, Aidan Fortier, Ian C. G. Weaver, Tara S. Perrot

**Affiliations:** 1 Department of Psychology and Neuroscience, Dalhousie University, Halifax B3H 4R2, Nova Scotia, Canada; 2 Department of Psychiatry, Dalhousie University, Halifax B3H 4R2, Nova Scotia, Canada; 3 Brain Repair Centre, Dalhousie University, Halifax B3H 4R2, Nova Scotia, Canada

**Keywords:** chromatin plasticity, CRF, hypothalamus, maternal care, paternal effects, predator stress

## Abstract

Detecting past experiences with predators of a potential mate informs a female about prevailing ecological threats, in addition to stress-induced phenotypes that may be disseminated to offspring. We examined whether prior exposure of a male rat to a predator (cat) odor influences the attraction of a female toward a male, subsequent mother–infant interactions and the development of defensive (emotional) responses in the offspring. Females displayed less interest in males that had experienced predator odor. Mothers that reared young in larger, seminaturalistic housing provided more licking and grooming and active arched back-nursing behavior toward their offspring compared with dams housed in standard housing, although some effects interacted with paternal experience. Paternal predation risk and maternal rearing environment revealed sex-dependent differences in offspring wean weight, juvenile social interactions, and anxiety-like behavior in adolescence. Additionally, paternal predator experience and maternal housing independently affected variations in *crf* gene promoter acetylation and *crf* gene expression in response to an acute stressor in offspring. Our results show for the first time in mammals that variation among males in their predator encounters may contribute to stable behavioral variation among females in preference for mates and maternal care, even when the females are not directly exposed to predator threat. Furthermore, when offspring were exposed to the same threat experienced by the father, hypothalamic *crf* gene regulation was influenced by paternal olfactory experience and early housing. These results, together with our previous findings, suggest that paternal stress exposure and maternal rearing conditions can influence maternal behavior and the development of defensive responses in offspring.

## Significance Statement

The differential allocation hypothesis implies that animals can detect prior experiences of potential mates through variation in the behavior of that animal and then vary their own reproductive investment accordingly, yet little is known about its role in offspring development. The authors examine the effects of predator odor exposure in male rats on female partner preference and maternal care, and show sex-specific changes in juvenile play and anxiety-related behavior. Epigenetic regulation of hypothalamic *crf* in response to stress is also influenced by paternal experience, which is contextually dependent on the rearing environment. This argues that preconception paternal stress and housing can influence maternal care and the development of defensive behaviors in offspring.

## Introduction

In the rat, postnatal maternal behavior is a critically important part of the early nurturing environment with respect to neurobehavioral development of subsequent generations (for review, see Weaver, 2010, 2014). Anticipatory parental effects, for example, through detecting the predation risk experienced by a potential mate, may allow females to adjust maternal behavior in order to increase their own survival and/or to increase the survival of offspring by preparing the neonates for living in the forecasted environment where certain threats are present (Harris and Uller, 2009; Mousseau et al., 2009). Indeed, gestational predator odor exposure (OE) has been used previously to exert effects on offspring (Korgan et al., 2014; St-Cyr and McGowan, 2015), and when administered soon after parturition increases maternal behavior (McLeod et al., 2007; Mashoodh et al., 2009) and alters anxiety in adult offspring (Mashoodh et al., 2009). This is consistent with other literature showing that female rodents are capable of altering maternal behavior based on other environmental features of their mates, such as adolescent exposure to environmental enrichment (Mashoodh et al., 2012).

Observational studies have provided evidence for stable individual differences in two main forms of mother–pup interaction, licking/grooming (LG) and arched-back nursing (ABN) posture, over the first week of lactation (Stern, 1997; Champagne et al., 2003a). Maternal LG-ABN behavior during the first week of life is associated with the long-term programming of individual differences in the responsiveness of the hypothalamic–pituitary–adrenal (HPA) axis, anxiety-like and cognitive performance, and reproductive behavior in the rat (Weaver, 2011). As adults, the offspring of high LG-ABN mothers show decreased expression of corticotropin-releasing factor (CRF), in the paraventricular nucleus of the hypothalamus (PVN), and a lower corticosterone response to stress by comparison with adult animals reared by low LG-ABN mothers (Liu et al., 1997; Caldji et al., 1998; Francis et al., 1999). The offspring of low LG-ABN dams have increased DNA methylation and decreased acetylation of lysine 9 on histone H3 (H3K9ac) of the exon 1_7_ glucocorticoid receptor-α (GRα) promoter region, decreased NGFI-A transcription factor association, and decreased GRα expression (Weaver et al., 2004, 2007,2014); leading to disinhibition of CRF secretion and a higher corticosterone response to stress (Liu et al., 1997; Caldji et al., 1998; Francis et al., 1999).

In addition to maternal behavior, recent studies have demonstrated the effects of paternal age (Smith et al., 2009, 2013), obesity (Ng et al., 2010; Fullston et al., 2013), enrichment (Mashoodh et al., 2012), and physiological/psychological stress (Franklin et al., 2010; Dietz et al., 2011; Hoyer et al., 2013; Mychasiuk et al., 2013; Rodgers et al., 2013; Gapp et al., 2014; Wu et al., 2016) on offspring. These paternal effects could be disseminated via sperm (Dias and Ressler, 2014), facilitated by sperm miRNA (Rodgers et al., 2013, 2015; Gapp et al., 2014), but maternal behavior may also propagate these effects (Mashoodh et al., 2012). The differential allocation hypothesis suggests that the dam can detect prior experiences of potential mates through variation in his behavior and/or chemical cues, and then vary her own reproductive investment accordingly, including offspring-rearing strategies (Burley, 1988). For example, dams mated with males that had been reared in an enriched environment show increased LG-ABN behavior toward their offspring (Mashoodh et al., 2012). Consistent with this, we have shown that early rearing in seminaturalistic housing (SNH) has profound effects on offspring development—induced seizure severity and number of CRF-immunoreactive neurons were reduced in juvenile rats raised in SNH compared with offspring reared in standard housing (SH; Korgan et al., 2014, 2015). This raises the question of whether the effects of SNH on *crf* gene regulation and stress responsivity are propagated by variations in maternal behavior.

In the present study, we take advantage of the properties of predator cues and the ecological validity of predation threat to examine whether maternal behavior can be indirectly influenced by the prior predator experience of a mate. Herein, we examined the potential interaction of paternal predation threat and maternal environment on maternal behavior and the development of social and defensive responses in the offspring. Fear and anxiety-like behaviors were examined in the adolescent offspring, along with H3K9ac association and *crf* promoter activation in the PVN.

## Materials and Methods

### Animals and breeding

**Figure 1.**
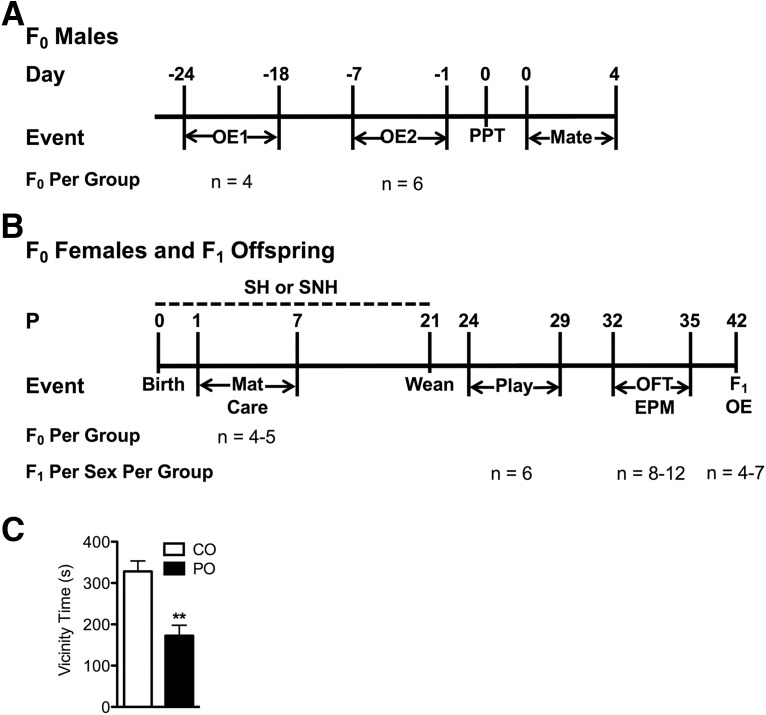
Experimental timelines and avoidance behavior in males during odor exposure. ***A***, Timeline of treatment procedures involving F_0_ males. During OE1, males were exposed to either PO or CO for 30 min, three times per day for 7 d beginning 24 d prior to mating. OE2 was conducted identically except that the CO and PO exposures began 7 d prior to mating. Males from OE1 and OE2 were subjected to a PPT using sexually receptive virgin, naive females. Within 12 h of the PPT, males were bred with different receptive naive virgin females. Following confirmed mating, males were removed, and females were left undisturbed until offspring were born. ***B***, Timeline of treatment procedures for F_0_ females and the F_1_ offspring. Birth was considered P0, and offspring were counted, sexed, and weighed before being transferred to either fresh SH or SNH, with biological mothers, until weaning. Maternal behavior (Mat Care) was scored for 1 h, five times per day for 7 d. At P21, all offspring were weighed, weaned, and placed in SH with a same-sex littermate. Play behavior was recorded in the home cage from P24 to P29, followed by exposure to the OFT and the EPM on P32 to P35. F1 OE took place on P42 with male and female offspring being exposed to either PO or CO for 30 min and then killed. Sample sizes are provided for both the F_0_ and F_1_ groups. ***C***, Avoidance behavior in F_0_ male rats was significantly increased in those exposed to PO relative to those exposed to CO during OE. Data are expressed as the mean ± SEM. **p ≤ 0.005, PO different from CO.

Thirty-eight Long–Evans hooded rats, 20 males and 18 females (purchased from Charles River Laboratories) at ∼60 d old were used for first generation (F_0_) testing and breeding. All rats were housed in same-sex pairs and given 1 week to acclimate prior to the beginning of the experiment. Rats were housed in a colony room under a 12 h reversed light cycle (lights off at 9:30 A.M.). Temperature in the colony room was maintained at 21 ± 2°C. Rats were caged in SH, which consisted of polypropylene cages (47 × 24 × 20.5 cm) with wire lids, containing pine shavings for bedding (Hefler Forest Products) and a black polyvinyl chloride (PVC) tube (length, 12 cm; diameter, 9 cm), unless housed in SNH (see below). Both rat chow (Purina Lab Chow) and tap water were supplied *ad libitum*. When breeding occurred, as described below (Fig. 1A), one male and one naive female determined to be in estrus were housed together for 5 consecutive days. Pups remained with the dam until weaning (day 21; Fig. 1B), at which time the offspring were rehoused with a same-sex littermate. All experimental procedures were performed in accordance with the guidelines of the Canadian Council on Animal Care and were approved by the Dalhousie University Committee on Laboratory Animals.

### Paternal stress exposure

The timeline of the described experimental procedures is shown in Figure 1, *A* and *B*. Following the 1 week acclimation period, the paternal OE trials began. Male cage mates were randomly assigned to one of the following two experimental conditions: paternal stress [predator odor (PO); *n* = 10] or control odor (CO; *n* = 10). Trials were 30 min in duration and took place at approximately 9:30 A.M., 12:30 P.M., and 3:30 P.M. for 7 consecutive days. Males were transported from the colony room in covered transport cages to a designated testing room, where odor exposure trials took place under red light. Trials were performed in a clean Plexiglas test cage (60 × 27 × 35.5 cm) with a white plastic floor and a clear Plexiglas lid with ventilation holes. For PO males, the odor stimulus was a piece of cat collar, ∼1 cm long, attached via an alligator clip to one end wall of the box, ∼5 cm from the top. The pieces of collar for the PO condition came from a collar that had been worn for at least 2 weeks by a reproductively intact domestic female cat housed communally in the Department of Psychology and Neuroscience at Dalhousie University. For CO males, the odor stimulus was a clean piece of collar. The testing of PO and CO conditions took place in different rooms and was performed according to an existing standard operating procedure designed to avoid cross-contamination (e.g., different gloves used and discarded in separate locations). All trials were recorded using a video camera for behavioral scoring. After each trial, rats were transported back to the colony room and placed back into their home cages. Videos were scored manually (by an observer blinded to the experimental conditions) to measure avoidance and anxiety-like behaviors, including the following: the frequency and duration of rearing (standing on hindpaws only, with or without leaning on the perimeter wall); grooming (≥2 s longer bouts that involve licking, nibbling, and combing-like actions of the fur); the duration of time spent within 10 cm of the wall possessing the odor stimulus (vicinity time); and the frequency of odor stimulus contacts.

### Partner preference test

**Figure 2.**
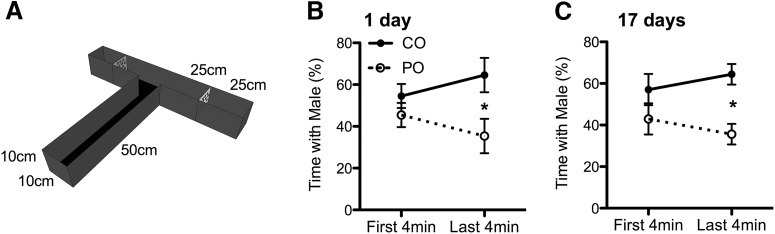
Use of a PPT to ascertain female preference for males previously exposed to PO relative to CO. ***A***, Schematic representation of the T-maze used for the PPT. Males were placed in the ends of arms confined by Plexiglas shields containing many holes. ***B***, ***C***, Female rats spent less time in the vicinity of PO-exposed males relative to CO-exposed males during the last 4 min of a partner preference test, both 1 and 17 d after the odor exposure had occurred in males. The percentage of time spent with males was calculated as the percentage of time spent with either a CO or PO male per total time spent with both CO and PO males. Data are expressed as the mean ± SEM. *p ≤ 0.05, PO different from CO.

Partner preference tests (PPTs) were performed either 1 or 17 d following the final odor exposure trial (Fig. 1A). In preparation for the PPT, sexually naive females were vaginally swabbed; only females in estrus were used. Six females were used in the PPT 1 d following odor exposure, and four females were used in the PPT 17 d following odor exposure. The PPT was performed using a T-maze (base: 50 × 10 × 10 cm; arms (×2): 50 × 10 × 10 cm; and a clear Plexiglas lid; Fig. 2A) containing two male rats and one female rat. Rats were transported to the testing room in covered cages. Trials occurred under red light and were recorded using a video camera positioned directly above the maze. Before the trial, the female rat was placed in the clean maze and allowed 5 min for habituation before being removed. Each 10 min trial was started by placing one female into the base of the maze. One PO male was placed in the end of one arm, and one CO male was placed in the end of the other arm. During the test, males were restricted to the arm ends and were separated by a clear Plexiglas sheet with multiple holes. The female was allowed to explore the entire maze. The total duration that the female spent inside an arm and oriented toward a male, along with the number of entries into each arm were recorded. For analysis, we calculated the percentage of the total time spent with each individual male per time spent with both males. Upon completion of a trial, the maze was cleaned with ethanol. Males were paired with sexually naive, receptive females within 12 h following the PPT for 5 d (Fig. 1A).

### Seminaturalistic housing

**Figure 3.**
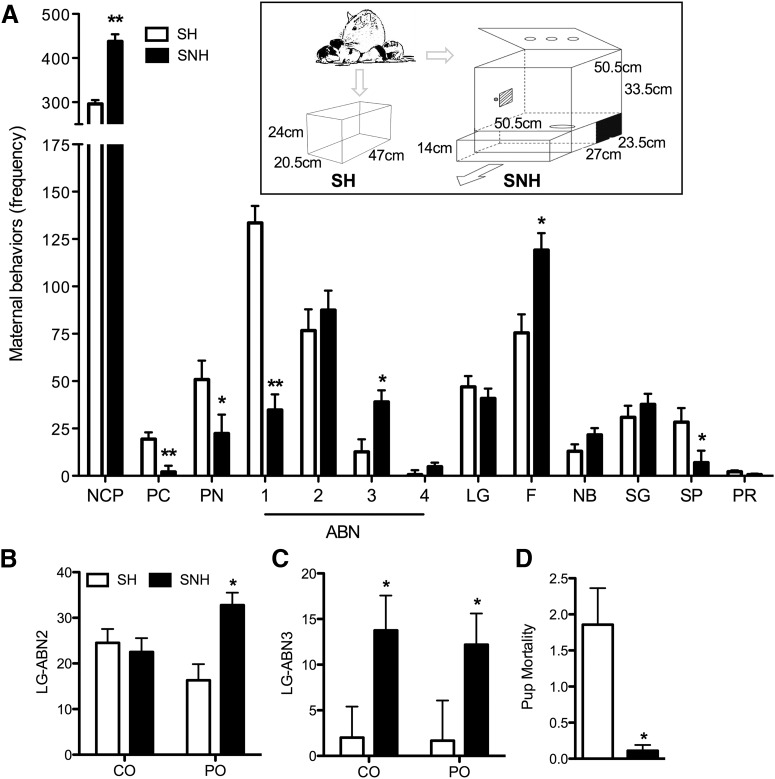
Maternal behaviors of females housed in SNH or SH raising offspring of mates that were exposed to either PO or CO. ***A***, Frequency of various maternal behaviors [PC, passive contact (with pups); ABN1, arched-back nursing 1 (blanket posture); 2, arched-back nursing 2; 3, arched-back nursing 3; 4, arched back nursing 4; NB, nest building; SG, self-groom (auto-groom); PR, pup retrieval] displayed by females housed in SH and SNH, collapsed across paternal condition. The inset shows schematics of the housing conditions; the SNH includes a lower burrow compartment (contained within a drawer that moves out to facilitate cleaning) and an upper section (containing food and water), with the two sections being connected by a hole (visible in the upper section). ***B***, The frequency of LG-ABN2 behaviors was significantly increased in dams raising offspring in SNH relative to those housed in SH, but only if offspring were from PO exposed males. ***C***, The frequency of LG-ABN3 behavior was increased in dams living in SNH relative to SH, regardless of paternal experience. ***D***, Living in SNH reduced pup mortality relative to living in SH. Data are expressed as the mean ± SEM. *p ≤ 0.05, SNH different from SH; **p ≤ 0.005, SNH different from SH.

Dams that were mated with CO and PO males were observed daily for pups once they reached gestational day 20, near the beginning of the dark cycle. Once the pups arrived [postnatal day 0 (P0)], the litter was sexed, counted, and weighed as quickly as possible to minimize disruption to the dams. Dams and litters randomly designated for the SNH condition (*n* = 10) were transferred to SNH cages on P0. Dams and pups in the SH condition (*n* = 8) were placed in clean, standard home cages. All dams and pups remained in their respective environments until the pups were weaned at P21. The SNH (Fig. 3A, inset) consisted of the following two sections: an upper section (50.5 × 50.5 × 33.5 cm) containing food and water available *ad libitum*; and a lower section (50.5 × 50.5 × 14 cm) filled with pine shavings and a PVC tube.

### Maternal care observations

The maternal behavior of dams was observed and scored daily in real time for 60 min at 8:00 A.M., 11:00 A.M., 1:00 P.M., 3:00 P.M., and 9:30 P.M. During each observation period, the frequency of the following behaviors was scored every 3 min, as in the studies by Champagne et al. (2003a) and Popoola et al. (2015): no contact with pups (NCP), which may or may not include self-grooming (SG), nest building (NB), or feeding behaviors (Fs); passive nursing (PN); arched-back-nursing (ABN), ranked as level 1 (ABN1; low blanket posture) and levels 2–4 (ABN2 to ABN4; high postures favorable for milk ejection); licking and grooming pups; separated pups (SPs); and pup retrieval (PR). The no-contact behaviors consist of a dam making no contact with her pups and often being accompanied by self-grooming behavior (licking, nibbling, and combing-like actions of the fur), nest building (changing the positioning or location of the pine shavings around the nest), and feeding behavior (nibbling at the feeder, consuming rat chow, or drinking water). PN was scored when the dam was on her side to nurse her pups or used the sides of the cage to support her while nursing. Blanket posture or ABN1 was observed when the dam was flat over the pups. ABN consisted of graded degrees of arching, levels 2–4, based on kyphosis or the bend of the knees and steepness of back arching of the dam. Separated pups were recorded when a dam had pups away from the nest, isolated or in small groups. Pup retrieval was the transfer of pups back into the nest.

### Offspring groups

The following four groups of male and female offspring were studied as juveniles and in periadolescence: CO-SH, father exposed to control odor and mother housed in standard housing (*n* = 12 males, *n* = 12 females); CO-SNH, father exposed to control odor and mother housed in seminaturalistic housing (*n* = 10 males, *n* = 10 females); PO-SH, father exposed to predator odor and mother housed in standard housing (*n* = 8 males, *n* = 8 females); and PO-SNH, father exposed to predator odor and mother housed in seminaturalistic housing (*n* = 8 males, *n* = 10 females). Each group contained two to four males and females from multiple litters, as follows: CO-SH, *n* = 4 litters; CO-SNH, *n* = 5 litters; PO-SH, *n* = 4 litters; and PO-SNH, *n* = 5 litters.

### Monitoring of offspring juvenile play

Pup play behavior observations began on PD24, and were conducted daily at approximately 11:00 A.M., 1:00 P.M., and 3:00 P.M. for 5 consecutive days. Observation sessions lasted 1 h, during which time an experimenter would record play behaviors observed in the home cage at a fixed interval of 3 min. Behaviors scored included social grooming (licking and/or chewing the fur of the conspecific, while placing forepaws on the back or the neck); “attack” behaviors, pouncing (play initiation, forepaws extended toward play partner, typically directed at neck, paws contact first), nose-to-nape attempted approach (nose approaches play partner's neck within 1-4 cm), nose-to-nape successful approach (nose contacts play partner's neck), and pinning (positioned over play partner with forepaws on partner); “defense” behaviors, full rotation (rolling supine, on back, to face play partner, interposing face/forepaws between attacker and nape), partial rotation (rolling toward supine to face play partner, but with at least one hindpaw on floor), upright defense (turn to face play partner from an upright position on hindpaws), and boxing (standing upright on hindpaws, forepaws extended toward play partner in pushing or swiping motions); and evasion (swerving or leaping away from play partner, fleeing; adapted from the study by Field et al., 2006).

### Anxiety behavior testing of juvenile offspring

Offspring anxiety behavior testing was performed on P32 to P35. Male and females (*n* = 8–12 per sex, per group) were tested in the open field arena and elevated plus maze (EPM). In both tests, each trial was recorded under red light, using a vertically mounted video camera, for future behavioral scoring. Pups were transported to and from the testing room in covered home cages, and the arena and maze were cleaned with 30% ethanol solution between each trial, as follows:

1. Open field test: the open field test (OFT) apparatus consisted of a solid, black Plexiglas square (79.2 × 78.9 × 35.0 cm), divided into 16, equal quadrants designated by nontoxic white paint on the maze floor. At the beginning of each 5 min trial, offspring were placed into the center of the maze. The behaviors scored for the OFT were as follows: line crosses (all four limbs crossing into a new quadrant), time in center (time spent in the four center squares, 25% of the total area); as well as freezing, grooming, and rearing (as defined for the EPM).
2. Elevated plus maze. The EPM apparatus was constructed of solid black Plexiglas, with two open arms (11.2 × 50.2 cm), adjacent to two closed arms (11.3 × 50.4 × 40.2 cm), elevated 40.0 cm from the floor. At the beginning of each 10 min trial, the rat was placed in the center platform of the maze (11.2 × 10.2 cm). Offspring behavior in the EPM trials was scored using video recordings and were as follows: line crosses (all four limbs crossing over the central platform); time in open arms (duration of time spent in open arms); time in closed arms (duration of time spent in closed arms); entries into open arms (all four limbs crossing into an open arm); entries into closed arms (all four limbs crossing into a closed arm); attempts into open arms (stretch-attend posture at the opening of an open arm, less than four limbs entering the arm); attempts into closed arms (stretch-attend posture at the opening of an closed arm, less than four limbs entering the arm); freezing frequency and duration (≥2 s period without movement, but not sleeping); grooming frequency and duration (≥2 s bouts that involve licking, nibbling, and combing-like actions of the fur); and rearing frequency and duration (standing on hindpaws only, with or without leaning on the perimeter wall).

### Acute stress exposure in periadolescent offspring

Seven to ten days after anxiety-behavior testing was completed, these same offspring were randomly assigned to either PO or CO exposure (*n* = 4–7 per sex, per group), identical to the protocol used for the paternal stress exposure (see above). The only variation in this protocol is that offspring were exposed only for one 30 min trial, followed immediately by killing.

### Killing and Tissue Collection

In the preparation of fixed tissue, animals were deeply anesthetized with Euthanyl (sodium pentobarbital, 60 mg/kg, i.p.) and then perfused transcardially with heparinized saline (30–60 ml), followed by paraformaldehyde (4%) in PBS, pH 7.4, for 15 min. After perfusion, all brains were removed and postfixed in the same fixation solution overnight at 4°C and then transferred to PBS-containing sucrose (20%) for 48 h. Tissue was frozen at −80°C until further processing. Whole brains were blocked and sectioned with a microtome. PVN-containing sections (coordinates with respect to bregma were −1.6 to −2.12 mm anteroposterior, 1.5 mm lateral from the midline, and −9.0 mm dorsoventral from the dura) were identified using the rat brain atlas (Paxinos and Watson, 1986), micropunched using a 20 gauge cannula (PlasticsOne), and stored at −80°C until used. To confirm the dissection site, serial coronal sections (20 mm thick) of the micropunched PVN tissue were cut on the microtome, were thaw mounted onto positively charged microscope slides, and were stored at −80°C. Slices were then stained with 0.25% DAPI (Roche Life Science) for 1 min and were mounted in PermaFluor Aqueous Mounting Medium (ThermoFisher Scientific). Images were acquired with a Zeiss Axio Imager Z2 fluorescent microscope (Carl Zeiss) and a high-resolution color digital camera using a 10× objective. At least four sections were examined per animal (see Fig. 6E).

### Chromatin immunoprecipitation

Chromatin immunoprecipitation (ChIP) assays (Crane-Robinson et al., 1999) were performed following the ChIP assay kit protocol (catalog #06-599, Upstate Biotechnology). Chromatin was immunoprecipitated from PVN micropunch samples using a rabbit polyclonal antibody against H3K9ac or normal rabbit IgG nonimmune antibody (both from Santa Cruz Biotechnology). One-tenth of the lysate was kept to quantify the amount of DNA present in different samples before immunoprecipitation (Input). Protein–DNA complexes were uncrosslinked by adding 20 μl of NaCl (5 m) to each sample (4 h, 65°C), followed by 10 μl of EDTA (0.5 m), 20 μl of Tris-HCl (1 m), pH 6.5, and 2 μl of PK enzyme (10 mg/ml; 1 h, 45°C). Following phenol-chloroform (0.5 v/v) extraction, the free DNA was ethanol (2 v/v, 95%) precipitated with 5 μl of tRNA (10 mg/ml) and resuspended in 100 μl 1× tris-EDTA buffer. The rat *crf* promoter region (−206 to 318, containing a cAMP response element) of the uncrosslinked DNA was subjected to PCR amplification (forward primer: 5'-TCAGTATGTTTTCCACACTTGGAT-3'; reverse primer: 5'-TTTATCGCCTCCTTGGTGAC-3'). For quantitative real-time PCRs, PCR mixtures (12.5 μl) containing the immunoprecipitated DNA, SsoFast EvaGreen Supermix (catalog #172-5203, Bio-Rad Laboratories), and 4 μm primer were loaded onto a 96-multiwell plate and covered with a seal (Bio-Rad). The thermocycler (CFX96 Touch Real-Time PCR Detection System, Bio-Rad Laboratories) protocol involved an initial HotStart enzyme activation cycle (2 min, 95°C, with a temperature transition rate set at 4.40°C/s), 40 cycles of denaturation (5 s, 95°C, with a temperature transition rate set at 4.40°C/s), and annealing (30 s, 60°C) with a temperature transition rate set at 2.20°C/s). A single fluorescence reading was acquired at the end of each elongation step. Triplicate average quantitative PCR (qPCR) cycle threshold (Ct) value for input (10%) samples: ∼24–26, with a fourfold to eightfold difference between qPCR Ct values of the H3K9ac antibody-immunoprecipitated samples (qPCR Ct value, ∼29–31) or negative control IgG nonimmune antibody-immunoprecipitated samples (IgG; Ct value, ∼34 or not detected after 40 cycles). The specificity of the amplified PCR products was assessed by performing a melting curve analysis cycle after the PCR amplification (5 s, 95°C, with a temperature transition rate set at 4.40°C/s; 1 min, 65°C, with a temperature transition rate set at 2.20°C/s) that terminated with a cooling step (30 s, 40°C, with a temperature transition rate set at 2.20°C/s). The fluorescence of the SsoFast EvaGreen dye bound to double-stranded amplified product declines sharply as the fragment is denatured. The melting temperature of this fragment was visualized by plotting the first negative derivative (dF/dT) of the melting curve on the *y*-axis and temperature (°C) on the *x*-axis. No primer-dimers were detected that interfered with the quantification of the PCR products. The Ct values of ChIP DNA fractions were normalized to the Ct value of the input DNA fraction for the same qPCR assay (ΔCt) to account for differences in chromatin sample preparation. Relative H3K9ac enrichment was measured by the 2^-ΔΔCT^ method, using the DNA fractions immunoprecipitated with IgG as the negative control (Livak and Schmittgen, 2001).

### Reverse transcription-qPCR analysis

Total RNA was isolated from PVN micropunch samples using the Arcturus Paradise Plus RNA Extraction and Isolation Kit (Life Technologies), which permits the recovery of high-quality RNA from a small number of fixed cells. Precipitated RNA was dissolved in RNase-Free H_2_O and quantified (∼274–335 ng of RNA/50 μl) with a Take3 Micro-volume Plate on an Epoch Spectrophotometer (BioTek). RNA integrity was confirmed using an Experion Automated Electrophoresis System and RNA StdSens chip (Bio-Rad). The RNA quality index value for all samples was >7.9 with low degradation. cDNA was synthesized in a 20 µl reaction volume containing 100 ng of total RNA, 40 units of Moloney murine leukemia virus reverse transcriptase (MBI), 5 µm random primer (Roche Molecular Biochemicals), a 1 mm concentration of each of the four deoxynucleotide triphosphates, and 40 units of RNase inhibitor (Roche Molecular Biochemicals). The mRNA was denatured (5 min, 70°C), the random primers were annealed (10 min, 25°C), and mRNA was reverse transcribed (1 h, 37°C). The reverse transcriptase was heat inactivated (10 min, 72°C), and the products were stored at −20°C. Rat PVN *crf* (NM_000756.1) heteronuclear RNA (hnRNA) was subjected to qPCR amplification (forward primer: 5'-TCAATCCAATCTGCCACTCA-3'; reverse primer: 5'-TAAGCTATTCGCCCGCTCTA-3'). To control for equal loading, the rat *ribosomal protein L13A* (*Rpl13A*; NR_073024) exon region was also subjected to PCR amplification (forward primer: 5'-ACAAGAAAAAGCGGATGGTG-3'; reverse primer: 5'-TTCCGGTAATGGATCTTTGC-3'). The *crf* hnRNA and Rpl13A amplifications were performed in parallel, using a 25 µl reaction mixture containing 1.5 µl of synthesized cDNA product and the SsoFast EvaGreen Supermix (Bio-Rad; Pfaffl, 2001). The thermocycler protocol involved an initial denaturation cycle (5 min, 95°C), 20–30 cycles of denaturation (30 s, 95°C), annealing/extension (45 s, 60°C), followed by a final extension cycle (5 min, 72°C) terminating at 4°C. The specificity of the amplification reaction was assessed by melt curve analysis and agarose gel electrophoresis of the PCR products. To control for equal loading between samples, the signal of the *crf* hnRNA was divided by the signal from the Rpl13A region amplified from the same sample.

### Statistical analyses

Differences between CO and PO males during OE were analyzed using an independent Student’s *t* test. For each group of males (1 and 17 d from OE), differences in time spent with CO and PO males by females in the PPT were analyzed using separate mixed-design ANOVA with paternal condition (CO, PO) as the between-subject factor and time block (first 4 min, last 4 min) as the within-subject factor. Maternal behavior was analyzed using two-factor ANOVA with paternal condition (CO, PO) and maternal condition (SH, SNH) as between-subject factors for each dependent variable. Offspring wean weight and behavioral test data were analyzed using linear mixed models. Sex, paternal condition, and maternal condition were used as between-subject factors, with litter treated as a nested factor for each dependent variable. Data from molecular end points were analyzed in an identical fashion with the addition of offspring odor exposure (F_1_CO, F_1_PO) as a fourth between-subject factor. Interactions were analyzed *post hoc* with simple effects analyses, with a Bonferroni correction. A threshold level of *p* < 0.05 was used to test for significance. The Statistical Package for the Social Sciences (SPSS) software was used for all statistical analyses.

## Results

### Predator odor exposure induces antipredator behavior in males and reduces partner preference in females

A summary of the research design is shown in Figure 1, *A* and *B*. To determine avoidance behavior in males in response to predator odor, we examined the time spent in proximity to the collar containing the odor. We then used a modified PPT as a proxy for sexual and social preferences of virgin age-matched females toward either PO or CO males (Fig. 2A, apparatus). Males exposed to PO spent significantly less time in the immediate presence of the collar compared with males being exposed to CO (*t*_(10)_ = 4.321, *p* < 0.001; Fig. 1C). There was no significant difference in the number of line crosses made by females during the PPT into arms containing CO-exposed versus PO-exposed males, either 17 d (*F*_(1,6)_ = 0.058, *p* = 0.818; CO: mean = 17, SE = 2.979; PO: mean = 16.5, SE = 2.979) or 1 d (*F*_(1,10)_ = 0.007; *p* = 0.935; CO: mean = 12.333, SE = 1.603; PO: mean = 12.167, SE = 1.603) after odor exposure (data not shown). During the last 4 min of the test 1 d after OE, we detected a main effect of PO treatment, with females displaying significantly less interest toward PO males compared with CO males (*F*_(1,10)_ = 5.131, *p* = 0.047; Fig. 2B). The effect of the predator experience of a male on female preference remained stable, lasting >2 weeks following the final predator odor exposure (*F*_(1,6)_ = 6.418, *p* = 0.044; Fig. 2C). These results suggest that the olfactory experience not only influences avoidance behavior in the male, but also stably increases avoidance behavior in females. Although correlations between paternal vicinity time during odor exposures and female preference were in the positive direction, indicating greater avoidance by females of males that had shown greater responsiveness during OE, these were not statistically significant (all males: *r* = 0.514, *p* = 0.088; PO males only: *r* = 0.436, *p* = 0.388).

### Seminaturalistic housing increases maternal care and interacts with paternal experience

Paternal stress (Gapp et al., 2014), maternal nurturing behavior (Weaver et al., 2004), and the context (Connors et al., 2015) of the early rearing environment have profound influences on postnatal development in the offspring. To examine these interactions, females were mated with PO and CO males and then raised their offspring in either SH or SNH, and the mother–infant interactions were monitored during the first week of postnatal life (Fig. 3A, inset, apparatus). We found no effects of, or interactions between, maternal and paternal conditions on litter size (*F*_(1,17)_ = 1.746, , *p* = 0.209), sex ratio of litters (*F*_(1,17)_ = 1.390, *p* = 0.260), or birth weight (*F*_(1,17)_ = 2.647, *p* = 0.128). Dams housed in the SNH behaved differently toward offspring relative to females housed in SH (Fig. 3A). SNH dams displayed significantly lower overall frequency of contact with their offspring (*F*_(1,17)_ = 18.730, *p* = 0.001), including the frequency of blanket posture (ABN1; *F*_(1,17)_ = 65.369, *p* < 0.001), passive nursing (*F*_(1,17)_ = 4.795, *p* = 0.047), passive contact (*F*_(1,17)_ = 24.446, *p* < 0.001), and pups separated from the litter (*F*_(1,15)_ = 4.815, *p* = 0.05), but showed significantly increased frequency of active arched-back-nursing (ABN3; *F*_(1,17)_ = 4.707, *p* = 0.049) and significantly more feeding behaviors (*F*_(1,17)_ = 10.933, *p* = 0.006). Interestingly, offspring from PO fathers received significantly more (*F*_(1,16)_ = 8.930, *p* = 0.011) maternal LG-ABN2 when raised in SNH relative to being raised in SH (Fig. 3B); this pattern was not observed for offspring from CO fathers. Maternal LG-ABN3 was increased in SNH conditions, regardless of paternal condition (*F*_(1,17)_ = 8.658, *p* = 0.011; Fig. 3C). Finally, pup mortality was significantly decreased in SNH conditions (*F*_(1,16)_ = 9.023, *p* = 0.011; Fig. 3D). These results, together with our partner preference findings, suggest that variation among males in their predator encounters may contribute to stable behavioral variation among females in courtship and maternal care, even when the females themselves are not directly exposed to a predator.

### Paternal odor exposure and SNH affect weaning weight and social behavior in juvenile offspring

**Figure 4.**
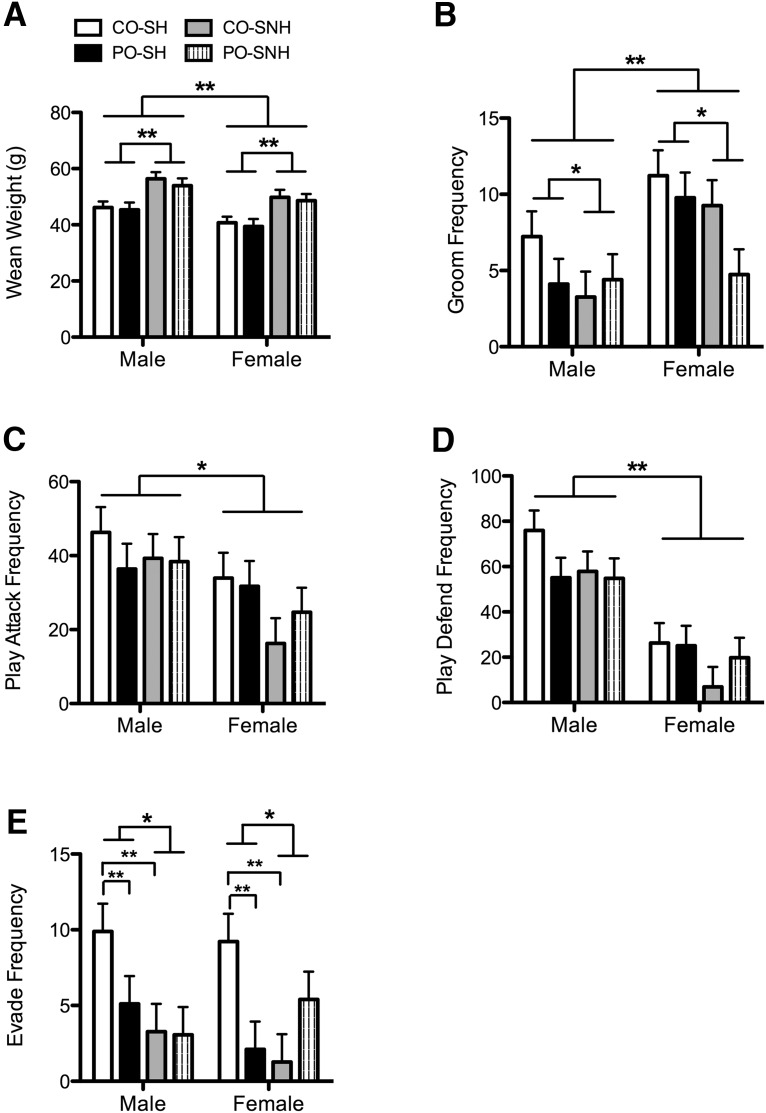
Weaning weight and social behavior of juvenile offspring raised by females housed in SNH or SH and sired by males exposed to either PO or CO. ***A***, SNH rearing significantly increased weaning weight in both male and female offspring, regardless of paternal experience, with males overall weighing more than females. ***B***, Increased social grooming occurred in female offspring, compared with male offspring, and was higher in offspring reared in SH relative to those reared in SNH. ***C***, Males engaged in significantly more play attacks than females. ***D***, Males also engaged in significantly more play defend behaviors relative to females. ***E***, Evading behavior in response to play attacks was decreased overall in offspring reared in SNH relative to SH, but more specifically, CO-SH-reared offspring evaded more than PO-SH- and CO-SNH-reared offspring. Data are expressed as the mean ± SEM. Difference between indicated groups: *p ≤ 0.05; **p ≤ 0.005.

To determine the extent of paternal stress effects and maternal nurturing behavior within the context of postnatal growth and social behavior development, we weighed the offspring at weaning and monitored play behavior in the home cage just after weaning (Fig. 4A–E). Males weighed more than females (*F*_(1,69)_ = 11.421, *p* = 0.001), and offspring raised in SNH weighed more than those raised in SH (*F*_(1,69)_ = 29.445, *p* < 0.001; Fig. 4A). Females groomed more than males (*F*_(1,39)_ = 11.602, *p* = 0.002), and offspring reared in the SNH groomed less than those reared in SH (*F*_(1,39)_ = 5.156, *p* = 0.029; Fig. 4B). Frequencies of play attacks (*F*_(1,39)_ = 7.711, *p* = 0.008; Fig. 4C) and defensive play behaviors (*F*_(1,39)_ = 44.194, *p* < 0.001; Fig. 4D) were greater in male offspring than in female offspring. Evading behavior in response to play attacks was decreased in offspring reared in SNH (*F*_(1,39)_ = 6.628, *p* = 0.014) relative to those reared in SH, but there was also an interaction between paternal stress experience and maternal rearing environment for this behavior (*F*_(1,39)_ = 9.322, *p* = 0.004; Fig. 4E). *Post hoc* comparisons revealed that CO-SH-reared offspring evaded more frequently than PO-SH offspring (*p* = 0.002) and CO-SNH offspring (*p* < 0.001). These findings suggest that postnatal growth and social behavior development are altered by housing environment, whereas preconception paternal predator odor exposure affects avoidance behavior in the periadolescent offspring.

### SNH affects the development of fear- and anxiety-like behavior in the offspring

**Figure 5.**
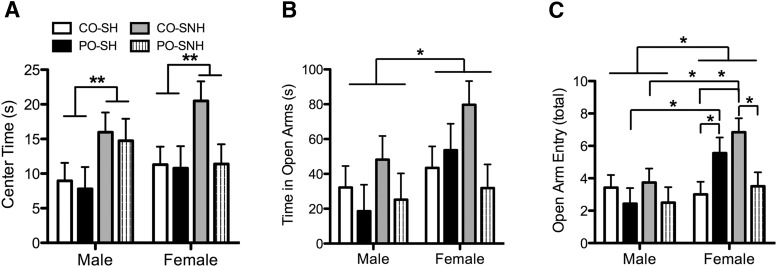
Development of fear- and anxiety-like behavior in the offspring raised by females housed in SNH or SH and sired by males exposed to either PO or CO. ***A***, Center time in the open-field test was increased (indicative of reduced anxiety-like behavior) in offspring raised in SNH relative to those reared in SH. ***B***, In the EPM, female offspring spent more time in the open arms relative to males. ***C***, Overall, in the EPM, female offspring displayed less anxiety (more frequent open arm entry) than males, and, in particular, females of either the CO-SNH or PO-SH group displayed reduced anxiety relative to other groups. Data are expressed as the mean ± SEM. Interaction: difference between indicated groups, *p ≤ 0.05; **p ≤ 0.005.

To determine the effects of paternal stress and maternal rearing environment on behavioral responses to stress in developing offspring, peripubertal offspring were exposed to OFT and EPM tests (Fig. 5A–C). In the OFT, offspring reared in the SNH spent more time in the center of the open field (*F*_(1,69)_ = 8.346, *p* = 0.005; Fig. 5A), regardless of paternal experience. In the EPM, females spent more total time in open arms (*F*_(1,69)_ = 4.635, *p* = 0.035; Fig. 5B) and entered open arms more frequently (*F*_(1,69)_ = 7.509, *p* = 0.008; Fig. 5C) than males. In addition to a significant interaction between maternal and paternal conditions (*F*_(1,69)_ = 5.986, *p* = 0.017), there was a significant three-way interaction among paternal condition, maternal condition, and sex for open arm entry (*F*_(1,69)_ = 5.112, *p* = 0.027; Fig. 5C). *Post hoc* comparisons revealed that PO-SH females made significantly more open arm entries relative to CO-SH females (*p* = 0.043) and PO-SH males (*p* = 0.024). Furthermore, CO-SNH females made more open arm entries than PO-SNH females (*p* = 0.008), CO-SH females (*p* = 0.002), and CO-SNH males (*p* = 0.013). Our findings show lasting sex-specific effects on stress response behaviors as a function of paternal stress experience and maternal rearing conditions.

### Effects of offspring predator odor exposure on their behavior and hypothalamic *crf* gene regulation

**Figure 6.**
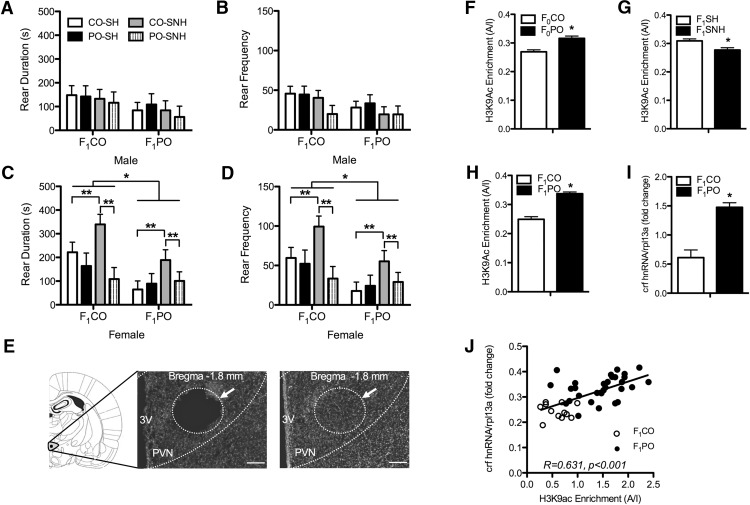
Effects of 30 min exposure to either CO (F_1_CO) or PO (F_1_PO) on behavior and hypothalamic *crf* gene regulation of offspring raised by females housed in SNH or SH and sired by males exposed to either PO (F_0_PO) or CO (F_0_CO). ***A***, Rearing duration was not significantly different in F_1_PO versus F_1_CO males, nor was it affected by paternal or maternal condition. ***B***, Likewise, rearing frequency was not significantly affected by short-term exposure to PO or paternal or maternal condition in males. ***B***, ***C***, Both rear duration (***C***) and rear frequency (***D***) were lower in F_1_PO-exposed females relative to F_1_CO-exposed females. Beyond the main effect, rearing frequency and duration were increased in all female offspring (exposed in the short term to CO or PO) of CO fathers and mothers housed in SNH. ***E***, Diagrammatic representation (left) and representative photomicrographs (10× objective) of DAPI-stained coronal sections showing the dissection site (white circle/arrow) in micropunched (middle) and intact (right) PVN tissue, in relation to the third ventricle (3V). ***F–H***, H3K9ac enrichment of the *crf* promoter in PVN was increased in F_0_PO relative to F_0_CO offspring (***F***), reduced in offspring raised in SNH relative to those raised in SH (***G***), and increased in F_1_PO offspring relative to F_1_CO offspring (***H***). ***I***, Levels *crf* primary transcript (hnRNA) in PVN were also increased in F_1_PO offspring relative to F_1_CO offspring. ***J***, Levels of H3K9ac enrichment of the *crf* promoter and hnRNA expression were positively correlated in F_1_CO and F_1_PO offspring. Data are expressed as the mean ± SEM. Difference between indicated groups, *p ≤ 0.05; **p ≤ 0.005. Scale bar, 200 μm.

We exposed the offspring of CO and PO fathers to the same threat experienced by the father preconception, to determine whether paternal olfactory experience predicted behavioral avoidance in the offspring (Fig. 6A–D). Although there was no effect of OE on male offspring behavior (Fig. 6A,B), female offspring exposed to predator odor reared for less time (*F*_(1,31)_ = 9.311, *p* = 0.005; Fig. 6C) and engaged in fewer total rears during the OE (*F*_(1,31)_ = 8.690, *p* = 0.006; Fig. 6D). Further, there was an interaction between paternal condition and maternal condition for both rear duration (*F*_(1,31)_ = 5.132, *p* = 0.031) and rear frequency (*F*_(1,31)_ = 5.355, *p* = 0.027), where CO-SNH females reared longer (Fig. 6C) and more frequently (Fig. 6D) relative to CO-SH females (*p* = 0.006 and *p* = 0.006, respectively) and PO-SNH females (*p* = 0.001 and *p* = 0.002, respectively). Given that seminaturalistic housing affected maternal care and fear- and anxiety-like behavior in the peripubertal offspring, animals killed within 30 min of odor exposure and tissue punches (Fig. 6E; for details, see Materials and Methods) were taken to measure hypothalamic *crf* gene expression and chromatin marks of gene regulation. Groups were collapsed across sex because no significant sex differences were observed in either the level of histone acetylation associated with the *crf* gene promoter region (*F*_(1,32)_ = 0.349, *p* = 0.559) or *crf* gene promoter activity (*F*_(1,32)_ = 0.110, *p* = 0.743), respectively. H3K9ac association with the *crf* gene promoter was significantly (*F*_(1,26)_ = 17.441, *p* < 0.000) increased in offspring of PO males (Fig. 6F) and decreased in offspring reared in the SNH (*F*_(1,26)_ = 7.898, *p* = 0.008; Fig. 6G). Moreover, offspring exposed to predator odor produced enhanced levels of H3K9ac association with the *crf* gene promoter (*F*_(1,26)_ = 58.934, *p* < 0.000; Fig. 6H), in addition to higher *crf* primary transcript (hnRNA) expression in the PVN compared with control odor-exposed animals (*F*_(1,32)_ = 29.024, *p* < 0.000; Fig. 6I). H3K9ac association and *crf* promoter activity were positively correlated (*r* = 0.631, *p* < 0.01; Fig. 6J), suggesting that stable differences in *crf* gene promoter acetylation drive hypothalamic *crf* gene expression and, possibly, fear- and anxiety-like behavior in the periadolescent offspring.

## Discussion

Here, we show interactive effects of paternal experience and maternal experience on anxiety-like phenotypes and associated stress-related molecular end points in offspring. Further, we have added a consideration for maternal care—a facet that had been lacking in previous paternal stress literature. Specifically, preconception PO experience in males stably influenced behavioral variation among female mates in partner preference and interacted with an enhanced maternal housing environment to affect maternal care and offspring social and defensive behavior. Paternal predator experience and maternal housing independently affected variations in histone acetylation and *crf* gene activity in response to an acute stressor in offspring.

Repeated exposure of prey to predators or their cues activates the HPA axis and initiates defensive behaviors (Mashoodh et al., 2008) that are long lasting, in part, through programming gene expression profiles supporting the neural circuitry of endocrine and behavioral responses to stress (Morrow et al., 2002; Wright et al., 2008, 2012; Roth et al., 2011). PO exposure in F_0_ males induced an avoidance phenotype similar to those found in past studies, in which we have noted that a similar repeated exposure paradigm results in increased baseline glucocorticoids and avoidance behavior (Mashoodh et al., 2008; Wright et al., 2008). In the present study, repeated exposure to PO resulted in an expected decreased preference of females for PO males relative to CO males. The results of our PPT suggest that the preference of the female for nonstressed males is based on the detection of a sensory or behavioral cue, rather than simply based on territory or copulative traits. In this initial investigation, we were interested mainly in comparing offspring of CO and PO males, and we did not investigate mating behavior directly. Thus, we are unable to determine whether female preference behavior incited males to increase male-directed courtship or whether elevated levels of male-directed courtship induced females to show preference behavior. While the directionality behind this pattern is unclear at this time, feedback and negotiations between males and females are important in mutual mate choice (Sheldon, 2000), and future investigations will likely reveal interesting effects from both sexes.

The relative influence of paternal versus maternal environments on maternal behavior has not been previously studied. Here, the contribution of maternal environmental effects was investigated by varying the housing conditions of females and their offspring. Past work shows that environmental enrichment of juvenile pups can reverse the maternal effects of low LG-ABN and decrease stress responsiveness in the adult offspring (Bredy et al., 2003, 2004). Previously, our laboratory has shown alterations in the severity of induced seizures and CRF-positive neuron numbers in the hypothalamus of juvenile offspring raised in SNH, suggesting stable and critical effects of this rearing environment on development (Korgan et al., 2014, 2015). Here, we continue to use juvenile and peripubescent animals to focus our investigations on effects during development. It is possible that our paternal or maternal conditions could have delayed pubertal development in pups, but, because behavioral testing was complete by P35, any delay (even in females who undergo puberty earlier) would likely not have impacted the behavioral testing. However, future studies should include measures of pubertal status.

For the first time, we show that offspring reared in SNH experience a different quality of maternal care. Similar to brief maternal separation (Liu et al., 1997; Connors et al., 2015), our SNH (which increased time away from pups) induced more active bouts of maternal care. SNH housing was associated with a marked decrease in blanket posture (ABN1), passive nursing, passive contact frequency, and pup mortality, contributing to an overall picture that SNH promotes more active, potentially higher-quality maternal care behavior. Interestingly, maternal LG-ABN2 behavior was increased by SNH, but only toward offspring that had been sired by a PO-exposed father. Thus, a dam may alter her behavior based on the past experience of her mate, but her own experience is at least as important for shaping her overall maternal care, and possibly outcomes in her offspring.

Social behavior and weight at weaning were affected mainly by sex of the offspring and rearing environment. At weaning, males were predictably heavier than females, and weight was increased in both SNH-reared males and females. Research is mixed on the effects of early enrichment on weight gain, likely dependent on the extent that enrichment is physical and/or social (Morley-Fletcher et al., 2003; Connors et al., 2015). Sex differences in offspring social behavior were similar to those found in other reports (Meaney, 1988; Pellis et al., 1997); females engaged in more grooming, while males were more active in play attack, and defensive and evasive behaviors. SNH rearing decreased grooming in male and female offspring. Maternal deprivation studies have identified deficits in sociality in offspring (Schneider and Koch, 2005; van Leeuwen et al., 2014). However, this difference in grooming could also be a manifestation of sex differences in play behavior, as male offspring engage in more attack grooming while females engage in more social grooming (Parent and Meaney, 2008). Future studies should distinguish between these two types of grooming behaviors. Further, in both sexes, CO-SH offspring displayed the most evasive behaviors in response to play attacks. This might suggest that both paternal odor exposure and SNH rearing are priming offspring for a more direct response to potentially threatening stimuli.

A complex picture emerged when examining the effects of maternal and paternal conditions on anxiety-related behavior in offspring. In the OFT, SNH-reared offspring spent more time in the center of the arena, similar to results using adolescent enrichment (Morley-Fletcher et al., 2003) and similar to offspring of high LG-ABN dams (Weaver et al., 2006). In the EPM, females showed higher amounts in time spent in open arms and in the number of open arm entries relative to males. Sex differences in anxiety behavior were not unexpected. The interaction among paternal experience, rearing environment, and sex in the EPM is more interesting. In female offspring only, being sired by a father that was exposed to predator odor or was being raised in seminaturalistic housing resulted in more open arm entries, indicating anxiolytic behavior in these females.

The effects of paternal stress experience on anxiety-related behavioral outcomes have revealed inconsistent findings in previous work, as some models have shown anxiolytic effects only in juvenile male offspring following paternal stress experience (Mychasiuk et al., 2013; Rodgers et al., 2013). Differences in offspring behavior could be related to the stressors used or the age of offspring at testing. The effects of maternal experience seem more straightforward, with previous research showing that the effect of prenatal stress on HPA axis function in offspring is sex dependent (McCormick et al., 1995; Brunton and Russell, 2010), and that variations of epigenetic marks, mediated by maternal care, are more pronounced in female offspring (Champagne et al., 2003b). Postnatal enrichment has also been shown to be more effective in female offspring (Welberg et al., 2006), which is consistent with our findings.

Exposing periadolescent female offspring to the PO paradigm resulted in the expected decrease in exploratory behavior, but male offspring exposed to PO did not show a reduction in activity relative to those exposed to CO, indicating a blunted behavioral response in males. The reason for this is unclear, but we have noted in other studies that patterns of behavioral response to PO are sex dependent (Mashoodh et al., 2012) and are not necessarily indicative of patterns associated with physiological responses (Mashoodh et al., 2008). Interestingly, females sired by a CO-exposed father and raised in SNH showed significantly higher levels of rearing behavior in response to CO and PO exposure relative to all other groups, which is suggestive of hyperactivity. We have observed previously that rearing behavior in female offspring is increased by manipulations that increased the levels of maternal care to which they were exposed (Mashoodh et al., 2009), suggesting a strong programming effect of maternal care on this particular behavior in female offspring.

Despite the sex differences noted in behavioral responses to odor exposure, PO exposure resulted in increases in H3K9ac association with the *crf* gene promoter in PVN as well as PVN *crf* hnRNA expression across both sexes, regardless of paternal or maternal condition. Interestingly, H3K9ac association with the *crf* gene promoter was increased in offspring sired by PO-exposed fathers but was reduced in those reared in SNH. Importantly, these modest changes in acetylation did not translate into significant changes in transcript levels—neither F_0_PO nor SNH rearing significantly altered *crf* hnRNA expression—whereas the dramatic increase of histone acetylation triggered by predator odor exposure in the offspring was associated with enhanced *crf* hnRNA transcription. These results suggest that while the presence of H3K9ac chromatin marks favor a transcriptionally permissive state, they are not sufficient to influence the transcriptional rate of the *crf* gene (i.e., the actual amount of target hnRNA or mRNA; Wang et al., 2009). The chromatin markings were also independent of sex, further suggesting that additional mechanisms are involved (Elliott et al., 2010). Beyond gene expression regulation, CRF function is mediated by CRF receptors (CRF_1_ and CRF_2_), which can be expressed in a sex-dependent manner (Howerton et al., 2014), and exert both additive and opposing influences on fear and anxiety behavior (Risbrough et al., 2004). Although further work is obviously required to fully explore the extent of these effects and to isolate the primary mechanism, our experiments do indicate the effects of both paternal and maternal history on key parameters of offspring stress responses.

Our findings provide the first evidence that variation in male predator experience influences partner preference by the female to produce stable alterations of chromatin structure and gene expression in the brain and behavioral responses to stress in the progeny. We showed an interaction between maternal condition and paternal condition on one measure of maternal care, but, overall, maternal behavior was more affected by maternal condition, and as such, future studies are needed to explore the mechanisms by which paternal behavior alters partner preference, exerts subtle effects on anxiety behavior, and impacts the epigenetic status of the hypothalamic *crf* promoter in the offspring. Clearly the gene–environment interaction is complex, especially as it pertains to early life programming and the transmission of stress-induced traits. Nevertheless, our findings provide a mechanism for the intergenerational transfer of stressful paternal experience to shape adaptive responses in the offspring, through differential allocation among females in both partner preference and maternal care. These findings may help to explain the immediate consequences of mothering style on pups, but the consequences are not necessarily self-perpetuating—such maternal effects appear to be dependent on the rearing environment early in juvenile development, resulting in stable alterations in phenotype of both the mother and her offspring.

## Synthesis

Reviewing Editor: Liisa Galea, University of British Columbia Decisions are customarily a result of the Reviewing Editor and the peer reviewers coming together and discussing their recommendations until a consensus is reached. When revisions are invited, a fact-based synthesis statement explaining their decision and outlining what is needed to prepare a revision will be listed below. The following reviewer(s) agreed to reveal their identity: John Howland, Erica Glasper

This manuscript examined the interaction of paternal predator odor stress and the maternal rearing environment on offspring behavior (fear, anxiety) and hypothalamic crf gene expression and regulation, by using maternal behavior as a potential mediator. Overall, the results are interesting; however, concerns about the small number of dams used in each condition and the design of the model dampened enthusiasm for the study somewhat. There were a number of statistical, design and interpretation issues that need to be addressed.

Methods and Design.

1.Please provide a description of the PPT test was mating allowed and if so were mating behaviors recorded and was there a difference. During the breeding period how long were CO and PO males paired with females? (there is some information in timeline but not in the actual methods section). Were mating tests recorded for analysis of mating behavior? Did CO or PO alter sexual performance? Or was pregnancy success rate altered between CO and PO males?

2.Were the litters culled? Were the numbers of male and female offspring equal in each of the litters? If not did these affect maternal behavior and/or other parameters in offspring?

3.How many males and females were used per litter in the offspring testing? Ie max 1-2?

4.Why were fewer males than females used for breeding? (Line 100-101)

5.How many female rats were used for the partner preference test?

6.Lines 212-213: Why were offspring tested between PD32 and PD35 if adult behavior was being assessed? Rats typically reach sexual maturity in a sex-dependent fashion, with females maturing earlier (PND32-35) and males maturing later (PND45-48). Sexual maturity marks the start of adolescence and not adulthood. Young adulthood is typically between 60 and 90 days of age.

7.As male and female rats were tested during puberty and as noted above there are sex differences in timing of puberty some indices of puberty need to be added to show that there were no differences in pubertal timing that could have affected the test results in the offspring. So for example if paternal PO experience delays puberty perhaps this is the cause of the differences in offspring seen in the present study.

8.The PPT was done either 1 or 17 days following OE ( olfactory experience?) and thus the PO males were mated at different times post exposure. Given there are only 8 PO males It is not clear if time since predator odor exposure influenced the results or was considered.

9.Were the PO and CO males housed together or per group after olfactory experience or one of each in a cage?

Statistics

10. All the reviewers felt there were significant concerns regarding the small number of litters used in each condition which needs to be addressed. While the authors included litter as a factor in the initial analyses they performed (lines 189-194), it is not known whether they had enough power to detect litter effects if they in fact exist (the authors only report their risk of a Type I error and not risk of a Type II error). These concerns have been raised previously (see, for example, Lazic 2013 Science 340:811) and must be dealt with directly in the present manuscript. Furthermore it may be important to explore the idea of using litter in the overall ANOVA as a nested design. It appears that 8 PO and 8 CO males were used but then 4 of these would be in the SH vs SNH groups and with the 1-17 days difference in PPT testing (exposure occurred on GD-24 or GD -7) this suggests that the n per group was quite small (n=2 litters?).

11. The statistical methods employed as described on page 14 are hard to discern. If sex differences were a primary outcome why were two separate analyses conducted - one with sexes treated separately and then together (this would result in 'double dipping'). In addition why are only main effects of sex important to examine, what about interactions with sex as a factor. Some of the analyses appear to have used a repeated measures design - for example data in figures 1D and 1E( i.e. PPT).

12. Line 338: While simple main effects were used to analyse interactions were any other post-hoc tests used in the analyses? Tukeys? Sidak? Fishers? How did the authors control for multiple simple main effect analyses?

13. The behavioral results, as reported, do not coincide with the depiction of the data in the figures. This makes it very difficult to interpret the findings and completely comprehend the results. For example:

Lines 397-398 and Figure 3B: The authors describe that PO male offspring showed less social grooming than CO male offspring. However, CO SNH males have similar grooming frequencies to PO males (solid dark bar, gray bar, and striped bar). So the original seems to be driven by SH CO compared to PO males.

Lines 401-402: Please state that the decrease in grooming in PO-SNH females compared to CO-SNH females is not significant as the p=0.056 is a 'trend' but not significant.

Lines 402-403 and Figure 3C: It is stated that there are no effects of total play attacks, however, Figure 3C shows a main effect of sex. Females display fewer play attacks than males. This should be clarified in the text.

Lines 454-456 and Figure 5F: The authors report that SNH decreased H3K9ac in males, however, Figure 5F does not depict lower H3K9 in the gray or striped bars (SNH groups) compared to the open and filled bars (SH groups). Further the authors report on lines 452-453 that H3K9ac enrichment was elevated in male offspring of PO males (figure 5F) but a decrease is seen under both SH and SNH.

Interpretation

14. It is unclear how the female rat "knows" the stress exposure history of the male rat it is forced to mate with. Given that naïve females were used for breeding, what is their experience with the range of potential mates and their range of stress?

15. Please be clear in introduction and discussion when describing the previous literature if the work was done exclusively in males or females. For example lines 53-58 - much of this work was done in males only and it is not always clear what effects, if any, there are on high/low LG-ABN in female offspring.

Minor Comments

1. Lines 3-4: The phrase "that may give rise to paternal effects in their offspring" is unclear and could benefit from rephrasing.

2. Lines 7-10: The description of the results of paternal stress on maternal behavior, as a result of housing, is not clearly described here. Combining the results in one sentence is cumbersome. I recommend that this be spelled out in separate sentences.

3. Lines 68-69 and lines 63-64 - Sex differences in which direction?

4. Line 105 - define SH - Ie standard housing? Line 442 (and in timeline): What does "OE" refer to?

5.Lines 137-139: It is unclear when mating was performed? Did you only mate males after 1 day after the final odor exposure, only 17 days after the final odor exposure, or both? Also, if both post odor-exposure conditions were used, did the mating tests begin 12 hrs after either? Please provide clarity within your text.

6.Lines 143-144: Was partner preference testing performed in the same room where the predator odor testing was performed or where the cat collars were housed? If so, the smell of the different apparati may be interfering with the behaviorial outcome.

7.Lines 374-375: Please add a period after (Figure 2A) so that this concludes this sentence. Start the next sentence with "SNH dams".

8.Please provide the means and SE or SEM for the overall locomotor activity of females during the partner preference tests. The statistics are provided but the data should be also.

9.Lines 384-386: Please consider reworking this sentence. The end of the sentence presents the pup death data but the sentence is worded improperly. Consider presenting the pup death data in a new sentence.

10.Lines 391-396: Please separate the weaning weight data from the social behavior data. It can stand on its own. Or, add that to the section header so that the reader knows to find those data in that section.

11.Line 408: Please change the second > in the sentence to a dash mark, as you are describing the group CO-SNH).

12.Lines 409-413: Please conclude the sentence "Finally, there..." with a period after "(Figure 3E)" and begin the next sentence with "Post-hoc...".

13.The threshold for statistical significance (p-value) should be reported.

14.The figures should be of equal width and height, if possible

15.The timeline in Figure 1A is cumbersome and difficult to read follow. It could benefit from redesign, so that it is easier to follow. For an example of detailed timeline that is easy to follow, I recommend Feierstein et al 2010, Frontiers in Behavioral Neuroscience.

16.Figures 1B, 1D, and 1E are not referenced in the text until the results section. Figures 1 A and 1C are referenced early in the Materials and Methods Section. Separating these timeline and T-maze images into one figure (Figure 1) and the partner preference test results (new Figure 2) into an additional figure will help with clarity and flow of the manuscript.

17.Please provide a title (i.e., 1-day and 17-day) for Figures 1D and 1E.

18.Figure 2A: For clarity sake, please 1) remove ABN from ABN1 and 2) place it below a line that runs under 1, 2, 3, and 4 on the x-axis. This way, the reader will know that 1, 2, 3, and 4 are ABN categories.

19.Figure 5G: Please include the line of best fit in this scatterplot.

20.Figure Legend Lines 810-811: Please describe the observed findings in Figure 1B and not just provide a title for the graph. Line 811: Please remove the sentence "Partner preference behavior in female rats." Lines 822-824: Please describe the observed findings in Figure 2B-2D and not just provide a title for the graph. Lines 831-833: Please describe the observed findings in Figure 3A-3E and not just provide a title for the graph. Lines 836-837: Please describe the observed findings in Figure 4A-4C and not just provide a title for the graph. Lines 841-843: Please describe the observed findings in Figure 5A-5B and not just provide a title for the graph. Lines 846-849: Please describe the observed findings in Figure 5D-5G and not just provide a title for the graph.

21.Tables In the response to reviewers, the authors state that a summary table was included in the revised submission. Was this subsequently removed from this submission?
